# Dissociable Cortical and Subcortical Mechanisms for Mediating the Influences of Visual Cues on Microsaccadic Eye Movements

**DOI:** 10.3389/fncir.2021.638429

**Published:** 2021-03-11

**Authors:** Ziad M. Hafed, Masatoshi Yoshida, Xiaoguang Tian, Antimo Buonocore, Tatiana Malevich

**Affiliations:** ^1^Physiology of Active Vision Laboratory, Werner Reichardt Centre for Integrative Neuroscience, Tübingen University, Tübingen, Germany; ^2^Hertie Institute for Clinical Brain Research, Tübingen University, Tübingen, Germany; ^3^Center for Human Nature, Artificial Intelligence, and Neuroscience, Hokkaido University, Sapporo, Japan; ^4^Department of Neurobiology, University of Pittsburgh Brain Institute, University of Pittsburgh, Pittsburgh, PA, United States; ^5^Graduate School of Neural and Behavioural Sciences, International Max-Planck Research School, Tübingen University, Tübingen, Germany

**Keywords:** superior colliculus, frontal eye fields, primary visual cortex, brainstem omnipause neurons, visual attention, microsaccades, fixational eye movements, visual selection

## Abstract

Visual selection in primates is intricately linked to eye movements, which are generated by a network of cortical and subcortical neural circuits. When visual selection is performed covertly, without foveating eye movements toward the selected targets, a class of fixational eye movements, called microsaccades, is still involved. Microsaccades are small saccades that occur when maintaining precise gaze fixation on a stationary point, and they exhibit robust modulations in peripheral cueing paradigms used to investigate covert visual selection mechanisms. These modulations consist of changes in both microsaccade directions and frequencies after cue onsets. Over the past two decades, the properties and functional implications of these modulations have been heavily studied, revealing a potentially important role for microsaccades in mediating covert visual selection effects. However, the neural mechanisms underlying cueing effects on microsaccades are only beginning to be investigated. Here we review the available causal manipulation evidence for these effects’ cortical and subcortical substrates. In the superior colliculus (SC), activity representing peripheral visual cues strongly influences microsaccade direction, but not frequency, modulations. In the cortical frontal eye fields (FEF), activity only compensates for early reflexive effects of cues on microsaccades. Using evidence from behavior, theoretical modeling, and preliminary lesion data from the primary visual cortex and microstimulation data from the lower brainstem, we argue that the early reflexive microsaccade effects arise subcortically, downstream of the SC. Overall, studying cueing effects on microsaccades in primates represents an important opportunity to link perception, cognition, and action through unaddressed cortical-subcortical neural interactions. These interactions are also likely relevant in other sensory and motor modalities during other active behaviors.

## Introduction

Vision is a particularly important sensory modality for primates, and it is processed in early visual brain areas by magnifying the neural representation of the tiny foveal region of the retinal image (Rovamo et al., 1978; Dow et al., 1981; Perry and Cowey, 1985; Azzopardi and Cowey, 1996; Chen et al., 2019). This form of neural specialization creates a need to closely coordinate vision and eye movements, the latter being particularly important to sequentially align the foveal retinal image with different salient and/or behaviorally-relevant targets. As a result, foveating eye movements (most typically, saccades) represent one of the most obvious forms of visual selection mechanisms, and a plethora of behavioral evidence confirms an almost-obligatory link between visual selection and foveating eye movements (Schneider and Deubel, 1995; Deubel and Schneider, 1996; Awh et al., 2006). Mirroring such a close relationship, cortical and subcortical brain areas that are critical for eye movement generation, such as the frontal eye fields (FEF) (Bruce and Goldberg, 1985; Bruce et al., 1985; Schall, 1991a, 2002; Schall and Hanes, 1993; Schall et al., 1995; Tehovnik et al., 2000), lateral intraparietal area (LIP) (Andersen et al., 1987, 1992; Barash et al., 1991a, b; Mazzoni et al., 1996), and superior colliculus (SC) (Cynader and Berman, 1972; Updyke, 1974; Wurtz and Albano, 1980; Sparks and Nelson, 1987; Munoz and Wurtz, 1995), all exhibit visual sensory responses as well movement-related discharge.

The need for visual selection also extends to cases in which we proactively attempt to dissociate our gaze position from the retinal image region that we wish to preferentially process. In this covert form of selection, visual processing capabilities of peripheral image regions can be enhanced or suppressed, depending on a variety of factors related to task demands. For example, a highly predictive cue presented at an upcoming peripheral target location may result in perception at that “cued” location being momentarily better than perception at competing image locations, in a so-called “cueing effect” (Posner, 1980; Nakayama and Mackeben, 1989; Cameron et al., 2002; Solomon, 2004; Pestilli and Carrasco, 2005; Carrasco, 2011). Historically, such covert orienting was studied exclusively under gaze fixation, with the assumption that small fixational eye movements do not influence peripheral visual sensitivity. There have been numerous reviews on the behavioral and neural properties of covert visual selection with this assumption (Bisley, 2011; Carrasco, 2011; Petersen and Posner, 2012; Anton-Erxleben and Carrasco, 2013; Krauzlis et al., 2013; Veale et al., 2017; Fiebelkorn and Kastner, 2019).

However, during gaze fixation, small saccades still occur, and it is now evident that they are functionally important for both vision and cognition. These eye movements, commonly called microsaccades, optimize eye position at the foveal target (Ko et al., 2010; Poletti et al., 2013; Intoy and Rucci, 2020), and they are also associated with foveal target selection processes (Poletti et al., 2017). This makes microsaccades functionally similar to larger saccades, in the sense that they serve the purpose of scanning visual image regions; in the case of microsaccades, the image regions just happen to be foveal. Interestingly, microsaccades also influence peripheral visual processing in intriguing manners. For example, microsaccades contribute to visual “refreshing” of retinal images whenever they occur (Martinez-Conde et al., 2000; Khademi et al., 2020). And, perhaps more importantly, microsaccades can alter peripheral visual sensitivity (Hafed, 2013; Chen et al., 2015; Tian et al., 2016; Lowet et al., 2018), in a manner similar to how visual sensitivity is affected during covert cueing paradigms. It is this last functional role of microsaccades that is particularly relevant for the present article: if microsaccades can influence peripheral visual sensitivity, are they systematically modulated in covert visual cueing paradigms? At the turn of the current century, two human behavioral studies were instrumental in answering this question (Hafed and Clark, 2002; Engbert and Kliegl, 2003). These studies uncovered a clear correlation between both the rate and direction of microsaccades and the onset and loci of peripheral events being covertly processed; there was also a relationship to behavioral performance improvements or impairments associated with cueing. These two studies contributed, at least in part, to a much renewed interest in microsaccades over the ensuing two decades. The net result was that a long lasting segregation between investigating covert visual selective mechanisms and ever-present fixational microsaccades had ended.

Nonetheless, strong debates quickly emerged, especially when it came to assessing a potential causal role for microsaccades in influencing peripheral visual performance (Hafed, 2013; Chen et al., 2015; Hafed et al., 2015; Tian et al., 2016; Lowet et al., 2018). Such debates can only be resolved, in our view, if sufficient knowledge about the neural mechanisms linking visual cueing and microsaccadic modulations is acquired. In this article, we describe the current state of the art concerning such mechanisms, and we hypothesize about the future directions that will likely develop. The picture that is emerging is one of an interesting dissociation between contributions of cortical and subcortical visual and motor circuits. Most intriguingly, the evidence so far points to the importance of visual sensory processing even in classically-viewed motor areas deep in the brainstem, and this idea, in our opinion, has the potential to significantly advance our understanding of the physiology of active vision in primates.

### Scope of This Article

We focus on causal perturbation experiments investigating how the onsets of peripheral visual cues can modulate microsaccades. As a result, the main emphasis will be on non-human primate studies. This emphasis exploits the remarkable repeatability of cueing effects on microsaccades in these animals (Hafed et al., 2011; Hafed and Ignashchenkova, 2013), thus enabling neurophysiological experiments. We also relate the findings to computational models, which were also motivated by non-human primate studies (Engbert et al., 2011; Engbert, 2012; Hafed and Ignashchenkova, 2013; Peel et al., 2016; Tian et al., 2016).

In all of the evidence that we review, we emphasize what is perhaps the most intriguing aspect of the links between microsaccades and visual selection: the large spatial dissociation between microsaccadic eye movement endpoints, which are tiny and foveal, and the peripheral loci of cues, cue-induced neural activity, and/or cue-influenced perceptual performance, which are all much farther out in eccentricity. This dissociation can clarify interesting properties of visual-motor interactions in cortical and subcortical circuits, especially with respect to how readout of oculomotor maps may be realized for eye movements in general.

## Microsaccades and Visual Cues

**FIGURE 1:**
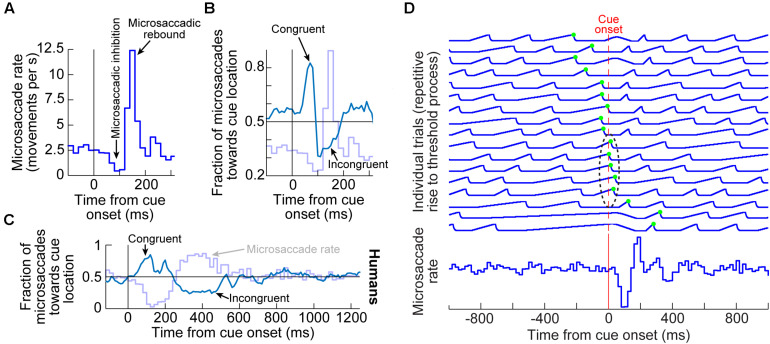
Systematic modulation of microsaccades after peripheral cue onsets. **(A)** Microsaccade rate from one monkey as a function of time from cue onset. In a baseline interval (e.g. before the cue), microsaccades come at a steady rate. Less than 100 ms after cue onset, microsaccade rate abruptly decreases (microsaccadic inhibition). A later rebound above baseline microsaccade rate then occurs, before a subsequent return to steady-state frequency. **(B)** The distribution of microsaccade directions relative to peripheral cue onset location is also time varying, and in a manner that is related to the microsaccadic rate modulations [the faint curve shows microsaccade frequency from **(A)** as a reference]. At the onset of microsaccadic inhibition, microsaccade directions are strongly biased toward the cue location (congruent microsaccades). Shortly afterward, at the onset of the rebound phase, microsaccades are strongly biased opposite the cue direction (incongruent microsaccades). **(C)** Human microsaccades show very similar modulations, but with slower temporal dynamics. **(D)** Mechanistically, the effects in **(A–C)** may reflect a resetting of ongoing microsaccade generation rhythms. Each fixation trial may be viewed as a repetitive rise-to-threshold process; a microsaccade is triggered at every threshold crossing (green dots indicate the time of the nearest microsaccade to stimulus onset). Cue onset resets the rise-to-threshold process, such that across trials, the modulations in **(A–C)** can emerge (bottom histogram for the case of microsaccade rate). Note how the trials highlighted with the black oval are trials in which cue onset comes too late to successfully reset the currently ongoing microsaccade rise-to-threshold process, resulting in very early microsaccades after cue onset. This theoretical framework suggests that cued-induced microsaccadic modulations depend on specific sensory and motor structures contributing specific components of the modulations in **(A–C)**, as we review in this article. **(A,B,D)** adapted with permission from Hafed and Ignashchenkova (2013); **(C)** adapted with permission from Tian et al. (2016).

Even though microsaccades were known to exist for many decades (Rolfs, 2009; Hafed, 2011), the impacts of peripheral visual cues on them only became documented in the present century. It is now known that the sudden onset of peripheral visual cues causes predictable microsaccadic modulations like those summarized in Figures 1A,B, based on data from rhesus macaque monkeys (Hafed and Ignashchenkova, 2013); very similar modulations also take place in humans (Figure 1C; Engbert and Kliegl, 2003; Galfano et al., 2004; Laubrock et al., 2005; Betta et al., 2007; Pastukhov and Braun, 2010; Engbert et al., 2011; Engbert, 2012; Tian et al., 2016).

In terms of microsaccade rate, the first modulation to occur is an abrupt cessation of microsaccade generation <100 ms after cue onset. This cessation is called microsaccadic inhibition (Figure 1A), and it is robust whether cues are behaviorally relevant or irrelevant, and whether cues are peripheral or foveal (Galfano et al., 2004; Laubrock et al., 2005; Betta et al., 2007; Rolfs et al., 2008; Hafed et al., 2011; Hafed and Ignashchenkova, 2013; Tian et al., 2016; White and Rolfs, 2016; Buonocore et al., 2017; Meyberg et al., 2017; Tian et al., 2018; Malevich et al., 2020). This inhibition also occurs with auditory stimuli (Rolfs et al., 2005, 2008), and it is generally similar to inhibition of larger saccades after abrupt visual onsets, in the phenomenon called saccadic inhibition (Reingold and Stampe, 1999, 2000, 2002; Buonocore and McIntosh, 2008; Edelman and Xu, 2009).

After a short microsaccadic inhibition interval of <100 ms, the second characteristic cue-induced modulation in microsaccade rate occurs. In this case, microsaccade rate increases to levels above the baseline pre-cue rate. This phenomenon is sometimes referred to as microsaccadic rebound, reflecting a rebound effect from the prior inhibition. In all cases with peripheral cues, the microsaccades are, by definition, too small to cause foveation of the visual onsets. Rather, they reflect an interaction between peripheral cue-induced visual activity and tiny microsaccade generation. Given the fact that many brain areas exhibit early stimulus-induced visual activity (and at approximately the same time as the onset of microsaccadic inhibition), the real question becomes which of these areas is causally most relevant for microsaccadic rate modulations.

In terms of microsaccade directions, even though the movements are not sufficiently large to foveate the appearing stimuli, their directions are still systematically related to them. Movements right before microsaccadic inhibition have directions that are highly congruent with the direction of the peripheral cues (Figures 1B,C). On the other hand, movements during the microsaccadic rebound phase are primarily directed in the opposite, cue-incongruent, direction (Figures 1B,C).

Thus, there is a microsaccadic direction oscillation (Figures 1B,C) that is unmasked by cue onset. The reason that we use the term “unmasked” is that behavioral and theoretical accounts have revealed that microsaccades tend to have temporal structure in terms of when they occur, and that such rhythmicity is associated with a general anti-correlation in direction between successive movements (Hafed and Clark, 2002; Abadi and Gowen, 2004; Gowen et al., 2007; Bosman et al., 2009; Hafed and Ignashchenkova, 2013; Tian et al., 2016). The role of peripheral visual cues in these accounts is, thus, akin to a phase resetting of the ongoing rhythms (Figure 1D), thus unmasking the direction oscillations (Engbert et al., 2011; Engbert, 2012; Hafed and Ignashchenkova, 2013; Tian et al., 2016; Bellet et al., 2017). Interestingly, phase resetting helps explain, at least partially, the strong microsaccadic rebound after microsaccadic inhibition: resetting results in a re-initiation of microsaccade generation processes; therefore, because it takes time to eventually trigger the movements after re-initiation, there will necessarily be a brief period of no microsaccades followed by a peak (Figure 1D). The peak time of the rebound reflects the approximate period of the microsaccadic rhythms, and subsequent peaks are washed out due to variability of inter-microsaccadic intervals.

We next describe the role of key cortical and subcortical brain structures that have been investigated with respect to such theoretical accounts. What one finds are dissociable impacts of different brain circuits to explain different aspects of the modulations. Perhaps most surprisingly, it is quite clear that microsaccadic inhibition, in particular, is not mediated by the most obvious candidate area repeatedly mentioned for it, the SC, as we now demonstrate. After demonstrating this, we will then relate the SC effects to effects mediated by other brain regions like the FEF, the primary visual cortex (V1), and the lower brainstem. By the end of the article, we will provide an integrative view of how we think all of the discussed brain areas complement each other in mediating the effects of Figure 1. This will provide a solid foundation for further exploring the functional role of microsaccades in covert visual selection mechanisms in the future.

## The Superior Colliculus (SC)

### The SC and Microsaccade Generation

The first investigations linking SC neural activity to microsaccades were not concerned with studying cue-induced microsaccadic modulations. However, these modulations, and the original two human studies (Hafed and Clark, 2002; Engbert and Kliegl, 2003), provided strong motivation to search for a potential causal role for the SC in microsaccade generation (Hafed et al., 2009, 2021; Hafed, 2011; Hafed and Krauzlis, 2012). Recordings in the rostral portion of the SC, in which small retinotopic eccentricities are represented (Cynader and Berman, 1972; Robinson, 1972; Krauzlis et al., 1997, 2000; Hafed et al., 2008; Hafed and Krauzlis, 2008; Chen et al., 2019), revealed microsaccade-related discharge (Hafed et al., 2009; Hafed and Krauzlis, 2012). Specifically, for a given subset of microsaccade directions and amplitudes, constituting a given rostral SC neuron’s movement field, the neuron emitted a strong burst of spikes starting right before microsaccade onset and peaking during the movement itself (Hafed et al., 2009; Hafed and Krauzlis, 2012). Moreover, reversible inactivation of the rostral region of the SC significantly reduced microsaccade likelihood (Hafed et al., 2009; Goffart et al., 2012).

Subsequent results related these motor properties of SC discharge to visual activity in the same rostral SC region (Chen et al., 2019). It was found that superficial neurons have foveal visual response fields and deeper neurons exhibit microsaccade-related movement fields (Chen et al., 2019). Interestingly, there are also visual-movement SC neurons for microsaccades (Willeke et al., 2019). Therefore, in the decidedly small realm of microsaccades, all of the classic properties of the SC in saccade generation were observed (e.g., visual responses in the superficial layers, and visual-motor and motor responses in the deeper layers). This represents an important development because it demonstrates a continuum between microsaccades and larger saccades (Hafed and Krauzlis, 2012), and a similar continuum between representing foveal and peripheral visual eccentricities (Chen et al., 2019). Such continua provide a good reason for investigating how peripheral SC activity during cueing may influence microsaccades.

In the past few years, discovering the role of the rostral SC in microsaccade generation became even more relevant for the context of the current article. Specifically, the similarity between microsaccades and saccades at the level of the SC led to a natural question (Hafed, 2011) on whether known peri-saccadic changes in visual perception, such as saccadic suppression of visual sensitivity (Zuber and Stark, 1966; Beeler, 1967; Riggs and Manning, 1982; Thiele et al., 2002; Lee et al., 2007; Bremmer et al., 2009; Krekelberg, 2010; Idrees et al., 2020) and saccadic distortion of visual space (Ross et al., 1997, 2001; Tolias et al., 2001; Zirnsak et al., 2014; Hartmann et al., 2017; Grujic et al., 2018), also take place around microsaccades. This was indeed the case. Around microsaccades, it was found that neural visual sensitivity can be enhanced or suppressed in several areas, including the SC and FEF (Bosman et al., 2009; Herrington et al., 2009; Hafed and Krauzlis, 2010; Chen et al., 2015; Bellet et al., 2017; Chen and Hafed, 2017). Most interestingly, sensitivity enhancement or suppression could occur at eccentricities far from the microsaccade endpoints (Hafed and Krauzlis, 2010; Chen et al., 2015). Because covert visual selection also involves alterations in sensitivity at eccentricities away from the fovea, this led to the intriguing possibility of linking peri-microsaccadic changes in perception (at far eccentricities) with changes that are normally attributed to covert visual selection. In other words, if microsaccades are not random during visual cueing (Hafed and Clark, 2002; Engbert and Kliegl, 2003; Laubrock et al., 2005), and if they are associated with changes in (foveal and peripheral) visual perception and neural activity when they do occur (Hafed, 2013; Chen et al., 2015), then could it be that performance changes in classic covert cueing paradigms are simply mediated by peri-microsaccadic changes in vision (Hafed, 2013; Hafed et al., 2015; Tian et al., 2016)? Such a hypothesis turned out to be entirely sufficient to account for some of the most classic cueing effects in the literature (Tian et al., 2016), but it was naturally controversial. To best assess how far such a suggestion could go in accounting for covert visual selection mechanisms, it became necessary to investigate the neural bases for cue-induced microsaccadic modulations, and the SC was the first natural place to look.

### The SC and Cueing Effects on Microsaccades

The key aspect of peripheral cueing effects on microsaccades is the spatial dissociation between cue locations and movement endpoints: small microsaccades are influenced by visual onsets having eccentricities that can be more than an order of magnitude larger than the movement amplitudes (Figure 1). Therefore, to understand the impacts of peripheral cueing on microsaccades, it was important to consider peripheral, rather than foveal, SC activity. The question, therefore, became whether peripheral SC activity that is induced by cue onsets (e.g., cue-driven visual bursts) is causally necessary for influencing cue-induced microsaccadic modulations.

To answer this question, Hafed et al. (2011) first relied on an established attentional cueing task used previously with monkeys (Lovejoy and Krauzlis, 2010). The task involved four placeholder rings appearing around the fixated point, in each of the four display quadrants. One of these rings was a color singleton, acting as the cue to covertly select a location for a subsequent perceptual discrimination. Since color singletons pop out in the SC representation of the visual image, resulting in higher activity at the singleton’s location (White et al., 2017a, b, 2019), it was expected that the onset of the color singleton was associated with differential peripheral spatial activation in the SC topographic map. It was, therefore, expected that microsaccades would be modulated after cue onset in this task (in a manner similar to Figure 1), and this was indeed the case (Hafed et al., 2011).

**FIGURE 2:**
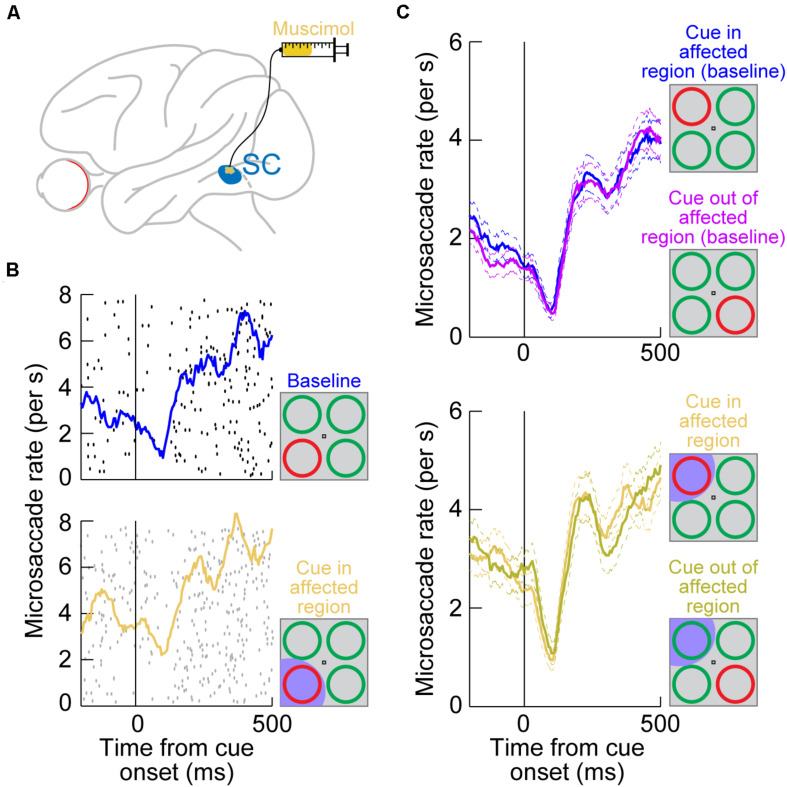
Reversible inactivation of the SC does not influence microsaccadic rate modulations after peripheral cue onsets. **(A)** Injection of the GABA agonist muscimol into a restricted region of the SC topographic map. The injection was intended to affect only an extra-foveal representation of the SC, such that microsaccade-related neurons in the foveal zone (Hafed et al., 2009; Hafed and Krauzlis, 2012; Chen et al., 2019; Willeke et al., 2019) were largely not affected. Rather, it was the representation of the location of a peripheral visual cue that was targeted [see the bottom schematic in **(B)**] (Hafed et al., 2013). **(B)** Microsaccade rate in a cueing task from a sample session before SC inactivation (top) and after inactivation (bottom). The task consisted of the onset of a color singleton ring as the cue in an attentional task (Lovejoy and Krauzlis, 2010); see schematics on the right. In this session, the cue appeared in the bottom left quadrant of the display relative to fixation position. Each black or gray dot indicates the onset time of a microsaccade relative to cue onset (different rows represent different trials), and all microsaccades toward or opposite the cue quadrant are shown. The colored curves show microsaccade rate estimates in each block. In the bottom panel, the SC representation of the lower left quadrant of the display was inactivated (shaded in the bottom schematic); that is, it was the representation of the singleton cue that was affected. Microsaccadic inhibition happened regardless of SC inactivation, and the overall rate modulation was similar with or without SC inactivation (Hafed et al., 2013). **(C)** Microsaccade rate from the same monkey across all sessions. The top panel shows rate modulations without SC inactivation when the cue was either in or outside of the region to be targeted by muscimol (opposite quadrants; see schematic insets on the right). Microsaccade rate modulations were identical, with strong microsaccadic inhibition shortly after cue onset. In the bottom panel, data from the same sessions are shown, but now with the SC inactivated in one quadrant of the visual display. Whether the cue was placed in the affected quadrant or opposite from it (see schematic insets on the right), microsaccadic rate modulations were similar. **(B,C)** Adapted with permission from Hafed et al. (2013).

The authors then reversibly inactivated a portion of the SC topographic map by injecting muscimol, a GABA agonist (Figure 2A; Hafed et al., 2013). The goal was to inactivate the SC representation of either the attended visual quadrant or the diametrically opposite one, but without affecting the rostral SC region where microsaccade generation commands are originated (Hafed et al., 2009; Chen et al., 2019). Once a portion of the SC map was rendered inactive, the same cueing task was run again with the peripheral cue (color singleton) being placed either inside the affected visual quadrant or in the diametrically opposite, unaffected, location.

Surprisingly, microsaccadic rate modulations after cue onset were unaltered by SC inactivation. For example, Figure 2B (top) shows microsaccade rate from an example session’s baseline data collected before muscimol injection. There was clear microsaccadic inhibition after cue onset, followed by a rebound. The very same modulation happened when the cue appeared in the affected quadrant (Figure 2B, bottom). Therefore, peripheral SC inactivation of cue-induced visual activity had no impact on the microsaccadic rate signature (Figure 1A). This conclusion was rendered even more concrete by inspecting the results from all inactivation sessions together (Figure 2C): whether the cue was placed inside the affected region of the display, in which cue-induced visual bursts were inactivated, or outside, the microsaccadic rate signature was the same. These results indicate that, contrary to expectations from models of microsaccadic and saccadic inhibition (Engbert, 2006; Rolfs et al., 2008; Bompas and Sumner, 2011; Engbert, 2012; Bompas et al., 2020), the SC is actually not causally involved in microsaccadic (or saccadic) inhibition.

**FIGURE 3:**
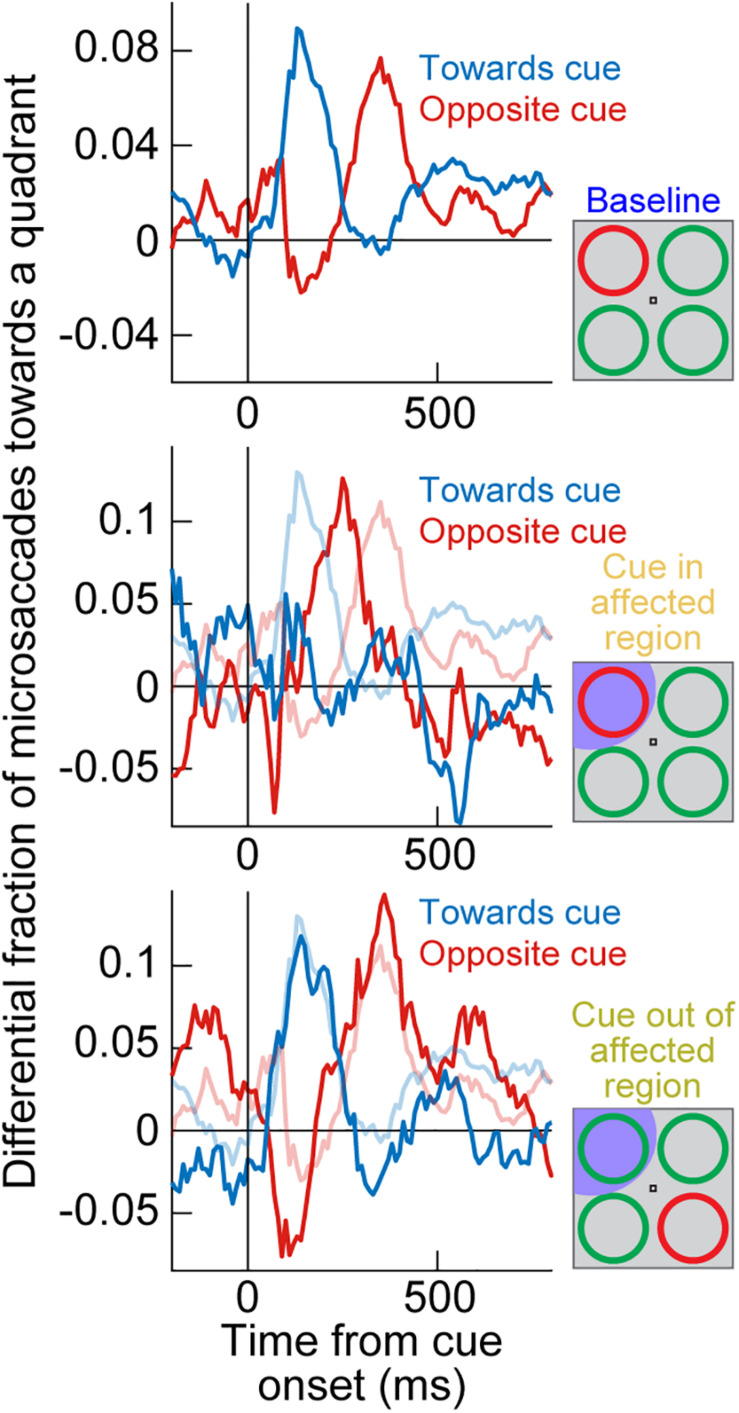
Reversible inactivation of the SC strongly influences cue-induced microsaccade direction oscillations by impairing the propensity for early cue-directed movements. Each panel shows a measure of propensity to generate microsaccades in a certain direction. In the top panel, the singleton cue onset, without SC inactivation, caused a clear microsaccade direction oscillation in the same monkey as in Figure 2. First, there was an increased likelihood of microsaccades toward the visual quadrant of the cue (blue curve), and then there was an increased likelihood of microsaccades toward the opposite quadrant (red curve). For simplicity, movements to the other two visual display quadrants (the least modulated by the cue) are not shown. This panel was adapted with permission from Hafed et al. (2011). In the middle panel, the cue appeared in the quadrant affected by SC inactivation (see shading in the schematic inset on the right). The early cue-directed microsaccades were massively reduced relative to baseline (blue curve), and oppositely directed microsaccades (red curve) came earlier than in baseline. The baseline curves from the top panel are shown in faint colors for comparison. When the cue was placed opposite the affected region (bottom panel), the direction oscillations were normal again, and very similar to the baseline data without any SC inactivation (faint colors). Therefore, cue-incongruent microsaccades after microsaccadic inhibition (e.g., Figure 1) are not affected by SC inactivation (even when they are still directed toward the affected quadrant, as in the bottom panel); only the earlier cue-congruent microsaccades are affected when the cue is in the impaired display region. The middle and bottom panels are adapted with permission from Hafed et al. (2013).

Where the SC was indeed causally relevant was in the cue-induced direction oscillations; these were strongly disrupted. In the same example monkey of Figure 2, a large array of behavioral trials from the same task had previously shown cue-induced microsaccade direction oscillations (Figure 3, top; Hafed et al., 2011). There was an initial period of microsaccades being congruent in direction with the cue location (blue curve) followed by a subsequent period of incongruent microsaccades (red curve; Figure 3, top); this is similar to the direction oscillations shown in Figures 1B,C. When the SC was now inactivated and the cue was placed inside the affected region of the display, the initial biasing of microsaccade directions toward the cued location disappeared (Figure 3, middle), and it was replaced by an earlier biasing of cue-incongruent movements. Thus, elimination of peripheral cue-induced visual bursts in the SC did not affect microsaccade rate modulations (Figure 2), but it did result in an imbalance of peripheral SC representations, which reduced the propensity to generate cue-congruent microsaccades in the early phase after cue onset. This result was the first causal evidence that microsaccade directions can be strongly influenced by peripheral neural activity, despite the fact that the microsaccade endpoints are much smaller than the eccentricities associated with such activity. It was now finally possible to pinpoint a clear mechanism for the directional effects of cueing on microsaccades. It was also possible to confirm a related hypothesis on peripheral cueing motivated by rostral SC investigations of microsaccade generation (Hafed et al., 2009).

Most interestingly, when the peripheral singleton cue was now placed opposite the inactivated visual field region (that is, in a portion of the SC map that was intact), the cue-driven microsaccade direction oscillations were largely unaffected (Figure 3, bottom). This result is particularly intriguing when one considers the late population of microsaccades directed opposite the cue (after microsaccadic inhibition had ended; see Figure 1). With the cue placed outside of the affected region, these late microsaccades were actually directed toward the region inactivated by muscimol, but they still happened as if the entire SC map was intact (Hafed et al., 2013). This suggests that the impairment of microsaccade directions with the cue being placed in the affected region (Figure 3, middle) was specific to cue-induced visual bursts occurring near the onset time of microsaccadic inhibition. When microsaccades happened later in time, microsaccades could still be generated in the direction of the affected SC region without any clear impairments (we demonstrate later how the FEF may be critical for these later microsaccades, explaining the lack of deficit with SC inactivation).

**FIGURE 4:**
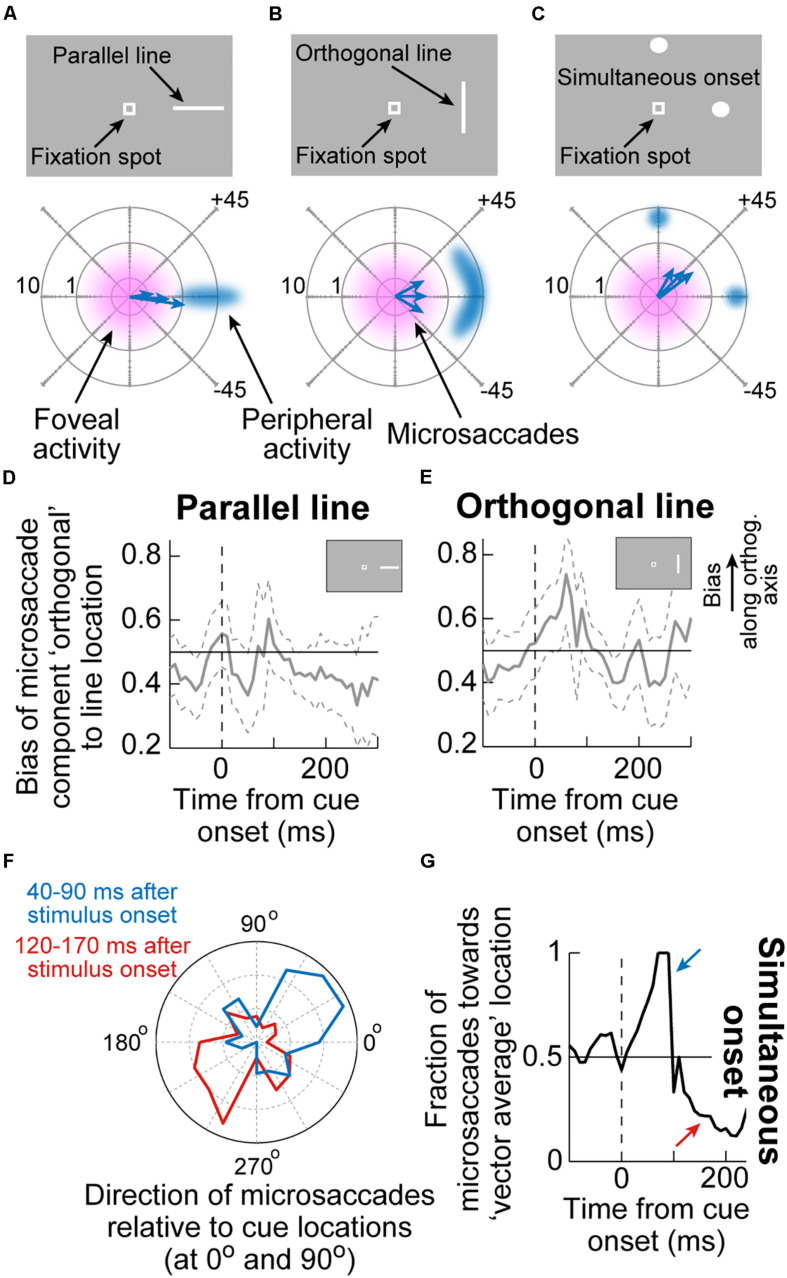
Sensitivity of cue-induced microsaccade direction distributions to the spatial layout of peripheral cue configuration. **(A)** If the peripheral cue is spatially distributed as a parallel line relative to cue direction (top), as opposed to just a spot, then early cue-congruent microsaccades would exhibit endpoint variance along the axis of the appearing line. The bottom schematic shows how microsaccade endpoints, despite being foveal and not reaching the peripheral stimulus location, are still cue-directed, but exhibit variance along the orientation of the line. Eccentricity in the bottom schematic is plotted on a logarithmic scale to visually magnify the small amplitudes. **(B)** If the peripheral cue is at the same peripheral location but is oriented as an orthogonal line instead, then early cue-congruent microsaccades (bottom schematic) would have vertical variance reflecting the spatial extent of the peripheral stimulus. **(C)** In the extreme of two simultaneous onsets, spatial readout from a map like that of the SC for saccades would predict microsaccades to neither of the stimuli (bottom schematic). **(D,E)** The time course of microsaccade orthogonal bias for the configurations in **(A,B)**. For a parallel line **(D)**, there is no orthogonal bias. However, for an orthogonal line **(E)**, early cue-induced microsaccades directed toward the peripheral stimulus have increased orthogonal variance, as in **(B)**. **(F)** For a simultaneous stimulus onset, like in **(C)**, early cue-induced microsaccades (40–90 ms) are directed toward the vector average direction of the two stimulus locations, consistent with saccadic readout of SC map activity (Lee et al., 1988; Glimcher and Sparks, 1993; Port and Wurtz, 2003; Katnani et al., 2012). Later microsaccades (120–170 ms) are opposite the vector average location. **(G)** Time course of the effects in **(F)**. Adapted with permission from Hafed and Ignashchenkova (2013).

An interesting additional implication of the results in Figure 3 is that they suggest that microsaccadic endpoint variance should be sensitive to the visual configuration of peripheral cues. That is, even though microsaccades never foveate the appearing peripheral cues, the early cue-congruent movements should still be sensitive to the spatial distribution of visual activation caused by the cues. Consider, for example, the two cue configurations in Figures 4A,B. In both cases, a peripheral stimulus appears to the right of fixation. However, in one case, the stimulus is a horizontal line (that is, with parallel visual activation to the axis connecting the line’s center to fixation), and in the other case, it is a vertical line (that is, with orthogonal visual activation to the axis connecting the line’s center to fixation). If peripheral visual bursts matter, then early cue-congruent microsaccades should exhibit endpoint variability that is parallel to the line’s orientation in both cases. This means that in the orthogonal case, early cue-congruent microsaccades would not only be directed toward the cue (Figures 1B,C), but their orthogonal endpoint variability should also now reflect the orthogonal orientation of the stimulus. Indeed, microsaccade endpoint variability turned out to be sensitive to such spatial stimulus manipulations (Figures 4D,E). In the extreme case, when the cue onset consisted of a simultaneous onset of two spatially segregated stimuli (Figure 4C), a prediction out of these results was that early cue-induced microsaccades should be directed toward neither stimulus, but toward the vector average location. This was again the case (Figures 4F,G; Hafed and Ignashchenkova, 2013), lending further credence to the notion that cue-induced microsaccade directions are particularly influenced by SC activity: even for large saccades (Findlay, 1982), readout of SC activity by the oculomotor system is known to result in vector averaging saccades when multiple simultaneous loci of neural activation exist on the SC topographic map (Lee et al., 1988; Glimcher and Sparks, 1993; McPeek et al., 2003; Port and Wurtz, 2003; Katnani et al., 2012; Vokoun et al., 2014).

**FIGURE 5:**
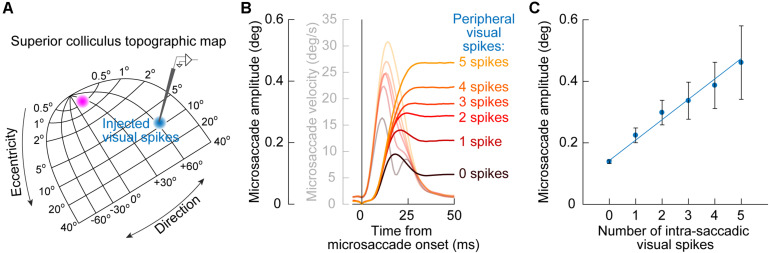
Quantitative link between microsaccade endpoint variability for early cue-induced microsaccades and the number of peripheral cue-induced SC visual spikes. **(A)** After cue onset, a visual burst is emitted in the peripheral SC representation (blue population of neurons on the SC topographic map). The timing of these visual burst spikes coincides with the timing of early cue-congruent microsaccades (e.g., Figure 1B). This might suggest that the impact of the SC on early microsaccade directions (as predicted from Figure 3) is mediated by readout, by downstream structures, of cue-induced visual spikes in the SC as if they were part of the simultaneously occurring movement spikes in the rostral SC (magenta population). **(B)** Consistent with this, cue-congruent microsaccades are larger in size than baseline microsaccades (Hafed and Ignashchenkova, 2013; Tian et al., 2016). More critically, the increase in size is deterministically related to the number of peripheral cue-induced SC spikes. The more “visual” spikes in a recorded peripheral SC neuron at the cued location, the larger the microsaccade. Faint colors also show microsaccade velocity profiles. **(C)** This relationship between cue-induced visual spikes in the SC and early cue-induced microsaccade amplitudes is linear for movements toward the cue (the great majority of movements shortly after cue onset). Thus, every spike of every active cue-driven SC neuron contributes to microsaccade endpoint variability. Adapted with permission from Buonocore et al. (2020b).

A further implication of the results of SC inactivation on early cue-influenced microsaccade directions (Figure 3) is that one can now attempt to establish a quantitative link between microsaccade endpoint variability and SC cue-induced visual bursts. Specifically, the timing of early cue-congruent microsaccades in Figures 1–3 is consistent with the timing of SC visual bursts (Buonocore et al., 2017, 2020b). If eliminating such visual bursts via SC inactivation diminishes the likelihood of cue-congruent microsaccades (Figure 3; Hafed et al., 2013), then this might suggest that these cue-congruent microsaccades reflect readout of the SC map under a very specific simultaneity condition: a microsaccade burst in the direction of the appearing cue in the rostral SC region, and a simultaneous visual burst in the periphery at the site representing the cue’s location (Figure 5A). If true, then there should be a measurable number of cue-induced visual spikes that are “injected” onto the SC map (when the cue appears) at the same time as the triggering of microsaccades (Buonocore et al., 2017, 2020b). This should “add” to the triggered movements and alter their size.

This idea was validated by measuring early cue-congruent microsaccade metrics: cue-congruent microsaccades were significantly larger in size than other microsaccades (Hafed and Ignashchenkova, 2013; Tian et al., 2016; Buonocore et al., 2020b). Importantly, there was also a quantitatively predictable relationship between the number of cue-induced visual spikes in the peripheral SC and the cue-congruent microsaccade amplitudes. To demonstrate this, Buonocore et al. (2020b) counted the number of visual spikes emitted by individually recorded peripheral SC neurons at the time of microsaccade triggering (Figure 5A). There was a linear relationship between the number of peripheral “visual” spikes and cue-congruent microsaccade amplitudes (Figures 5B,C): every single spike of every single visually-driven neuron contributed to the trajectory of early cue-congruent microsaccades (Buonocore et al., 2020b). Therefore, we now had a detailed mechanistic account of why early microsaccades after cue onset, near microsaccadic inhibition, are both directed toward the cue (Figure 1) and also larger in size (Figure 5).

### Summary and Outlook

The SC does not cause microsaccadic inhibition, unlike in previous modeling assumptions (Engbert, 2006; Bompas and Sumner, 2011; Bompas et al., 2020). However, cue-induced SC visual bursts do alter both the directions (Figures 3, 4) and amplitudes (Figure 5) of early cue-congruent microsaccades. Later cue-incongruent movements (i.e., during the microsaccadic rebound phase in Figure 1) are unaffected by SC inactivation (Figure 3, bottom). What, then, controls post-inhibition microsaccades? According to phase resetting hypotheses (Figure 1D; Hafed and Ignashchenkova, 2013; Tian et al., 2016, 2018), rebound microsaccades represent deliberate responses to the recently appearing and cognitively processed visual stimuli. From that perspective, cue incongruence would result from a willful attempt to avoid “breaking fixation” and overtly looking toward the cue (Tian et al., 2016, 2018). Indeed, with simultaneous stimulus onsets (Figures 4C,F,G), post-inhibition microsaccades were also directed opposite the vector average direction rather than toward or opposite either of the two stimulus locations, again suggesting compensation for an earlier reflexive tendency to look. In what follows, we describe how post-inhibition microsaccades are, therefore, particularly sensitive to FEF activity. This suggests a division of labor between cortical and subcortical influences on cue-induced microsaccades, and it paves the way for further discussions of V1 and lower brainstem involvements.

## The Frontal Eye Fields (FEF)

### The FEF and Microsaccade Generation

**FIGURE 6:**
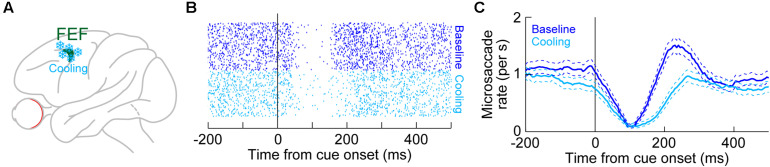
Reversible inactivation of the FEF, through cryogenic techniques, strongly influences microsaccade rate in the post-inhibition rebound phase. **(A)** Large portions of the FEF (either unilaterally or bilaterally) were reversibly inactivated by cooling of neural tissue. **(B)** Microsaccade onset times across trials (each row of rasters represents an individual trial) in baseline (blue) or with unilateral FEF inactivation (light blue). The task consisted of fixation with the onset of a peripheral spot in the affected hemifield. In baseline, there was expected microsaccadic inhibition shortly after cue onset, followed by a rebound in microsaccade likelihood. With FEF inactivation, microsaccadic inhibition still occurred, and with similar latency to baseline. However, the post-inhibition rebound was strongly impaired, resulting in an appearance of prolonged microsaccadic inhibition. **(C)** Microsaccade rate estimates for the data in **(B)**. Microsaccadic inhibition still clearly happened with or without FEF inactivation. However, microsaccadic rebound only happened when the FEF was intact (baseline). **(B,C)** Adapted with permission from Peel et al. (2016).

Unlike in the SC, there are currently no physiological recording data in the FEF demonstrating microsaccade-related neural discharge. However, the FEF is an important structure for mediating saccadic eye movements in general (Bruce and Goldberg, 1985; Schall, 1991b, 2002; Schall et al., 1995; Tehovnik et al., 2000; Sommer and Wurtz, 2001). Also, large-volume inactivations of the FEF, using cryogenic techniques (Figure 6A), significantly altered microsaccades (Peel et al., 2016). Specifically, unilateral inactivation of the FEF resulted in microsaccades becoming larger than normal. This effect was larger for contraversive microsaccades (that is, directed toward the visual hemifield affected by FEF inactivation) than for ipsiversive movements (Peel et al., 2016). Moreover, microsaccade kinematics were also altered, with both unilateral and bilateral FEF inactivation resulting in abnormally slower and longer-duration movements (Peel et al., 2016). In other words, the known main sequence relationship of peak velocity vs. amplitude (Zuber et al., 1965; Bahill et al., 1975) was shifted downwards by FEF inactivation. Interestingly, unlike in the rostral SC (Hafed et al., 2009; Goffart et al., 2012), unilateral FEF inactivation did not reduce baseline microsaccade rate in the absence of cueing (Peel et al., 2016). Only with bilateral FEF cooling was the overall microsaccade rate reduced.

These results were the first causal demonstration that FEF neural activity can influence microsaccades, likely by affecting both the SC and downstream brainstem oculomotor control circuitry. This is also consistent with the fact that unilateral FEF inactivation using the same cryogenic techniques also reduced the peak velocities of large saccades (Peel et al., 2014); also see related lidocaine and muscimol inactivation results in Sommer and Tehovnik (1997) and Dias and Segraves (1999). However, a causal impact of the FEF on microsaccades was also very interesting from the perspective of cueing effects on these eye movements, as we describe next.

### The FEF and Cueing Effects on Microsaccades

With unilateral large-volume FEF inactivation, microsaccadic rate modulations after the onset of a peripheral visual target still showed intact microsaccadic inhibition (Peel et al., 2016). In fact, even with bilateral FEF inactivation, microsaccadic inhibition was still largely unaffected. Therefore, like in the SC, the early microsaccadic inhibition phase of the microsaccadic rate signature (Figure 1A) was strongly resilient to impaired FEF activity.

However, unlike with the SC, FEF inactivation had a strong effect on microsaccadic rebound rate, and particularly with bilateral inactivation. For example, Figure 6B shows microsaccade times after cue onset in the absence of FEF inactivation (blue dots, top half of the panel) or during unilateral FEF inactivation (light blue dots, bottom half of the panel). The microsaccade rasters were very similar except during the rebound phase. This is also evident in Figure 6C, plotting the rate curves for the same data. Microsaccadic inhibition was unaltered by unilateral FEF inactivation, but there was an impairment in the generation of post-inhibition rebound microsaccades (Peel et al., 2016). This effect was almost doubled in size when the FEF was inactivated bilaterally (Peel et al., 2016), and bilateral inactivation also reduced overall microsaccade rates even in the baseline pre-cue interval as well (Peel et al., 2016). Once again, microsaccadic inhibition was largely unchanged by bilateral inactivation.

The effect of FEF inactivation on microsaccade rate during the microsaccadic rebound phase (Figure 6) is additionally interesting from the perspective of cue location. With unilateral inactivation, the cue could either appear in the affected hemifield (contralesional) or in the unaffected one (ipsilesional). In both cases, the rebound rate was reduced. This could reflect the fact that each of the right and left FEF might still contain a small component of ipsilateral visual field representation, and it is also consistent with the kinematic effects described above (Peel et al., 2016). Naturally, bilateral FEF inactivation also affected the rebound rate in both the right and left hemifields (Peel et al., 2016). Nonetheless, one could wonder whether the presence of a rate effect in Figure 6 and its absence in Figure 2 for the case of SC inactivation could reflect methodological differences between techniques. For example, the volume of tissue affected by cooling was putatively larger than that affected by muscimol injection (Hafed et al., 2013; Peel et al., 2016). Moreover, the SC (Chen et al., 2019) is more topographically organized than the FEF (Bruce et al., 1985; Sommer and Wurtz, 2000), allowing the authors of Hafed et al. (2013) to avoid, as much as experimentally possible, injection of muscimol into the rostral region where microsaccade-related discharge is found. Were the results of Figure 6, then, a technique artifact?

The authors of Peel et al. (2016) concluded that it is unlikely that methodological differences provided the full explanation for the microsaccade rate differences between SC and FEF inactivation. In fact, both of the above-mentioned methodological differences (which are inherent in the FEF experiments) should be expected to cause massive, non-specific effects on microsaccade rate. Rather, the effect of FEF inactivation was temporally specific (Figure 6), only affecting microsaccade rate in the rebound phase (Peel et al., 2016). Therefore, it is safe to conclude that the role of the FEF in cue-induced microsaccadic modulations is indeed critical for mediating the microsaccadic rebound modulation of Figure 1.

In terms of microsaccade directions, the overall results were slightly harder to interpret, especially because of a small offset in eye position caused by the FEF inactivation, and because of idiosyncratic biases of the monkeys even without inactivation (Peel et al., 2016). Nonetheless, there was a sufficiently clear pattern for the microsaccades occurring late after cue onset, in the microsaccadic rebound phase: the directions of these movements were affected the most by unilateral FEF inactivation. The specific effect was to temper the expected strong bias away from the cue. In other words, impairing the FEF also impaired the ability to bias late microsaccades away from the cue. Interestingly, this result was strongest when the cue had appeared in the affected hemifield, suggesting that the intact FEF somehow tags cue location for dealing with the microsaccades that come after microsaccadic inhibition, even when these microsaccades are cue-incongruent (Peel et al., 2016). Thus, the primary effect of FEF inactivation was to control the timing of microsaccadic deployment after microsaccadic inhibition, through modulation of the rebound phase (Peel et al., 2016). This is almost the opposite of the role of the SC in mediating cue-induced microsaccadic modulations, in which it was direction (and not rate) that was most affected, and earlier in time (Hafed et al., 2013).

Consistent with the above, changing a single parameter in the same theoretical model described in Figure 1D (Hafed and Ignashchenkova, 2013) could account for the influences of unilateral FEF inactivation. Specifically, after successful microsaccadic inhibition, these models (developed well before the FEF inactivation experiments) invoked a top-down facilitation factor for programming the first rebound microsaccade (that is, an increase in the slope of the rise-to-threshold process in Figure 1D). A conceptually similar top-down facilitation factor was also invoked in other models of cueing effects on microsaccades (Engbert, 2012). To model unilateral FEF inactivation, simply reducing this top-down facilitation factor was sufficient to replicate the results in Figure 6 (Peel et al., 2016). Interestingly, adding just an overall sluggishness for all microsaccades (that is, just reduced global drive) was also sufficient to account for the bilateral FEF inactivation effects of both reduced rebound but also reduced overall microsaccade rate in general (Peel et al., 2016).

### Summary and Outlook

A significant component of microsaccadic modulations after cue onsets is now relatively well understood in terms of dissociable causal contributions of the cortical FEF and the subcortical SC (Figures 2–6). What remains is to understand why microsaccadic inhibition is so resilient in the face of large perturbations of two key candidate areas for mediating it (the SC and FEF), and also to link the microsaccadic modulations to the bigger question of why microsaccades happen at all in the first place during cueing tasks. Answering the first question will complete the story of explaining all key components of the now-classic modulations seen in Figure 1, and answering the second question can help clarify the functional role of cue-induced microsaccadic modulations, particularly with respect to the hypotheses associated with peri-microsaccadic changes in covert visual selection performance that we alluded to earlier (Hafed et al., 2015).

## A Causal Behavioral Manipulation for Isolating Putative SC and FEF Contributions to Cueing Effects on Microsaccades

We will address the first question above shortly. For the latter, significant insight can be made when considering perturbation experiments that are not neural, but behavioral. Specifically, microsaccades, like other eye movements, ultimately alter the visual image impinging on the retina. Thus, even the simple act of fixating a tiny spot is an active visual-motor process, with microsaccades and ocular position drifts continuously shifting the visual position of the spot in the fovea. It therefore stands to reason that a visual error in the fovea (that is, a difference between where gaze is directed and where the target for fixation is in the retinal image) should be an important visual driver for microsaccades (Ko et al., 2010; Poletti et al., 2013). Indeed, microsaccades correct tiny foveal position errors, even during cueing tasks (Tian et al., 2016, 2018). In that regard, experimentally perturbing the natural active vision cycle for microsaccades, by experimentally eliminating expected modulations of foveal visual error as a result of microsaccade occurrence, should magnify and isolate the putative impacts of the SC (mediating reflexive cue-directed microsaccades) and FEF (mediating subsequent deliberate movements). Thus, so-called retinal-image stabilization experiments may be viewed as a behavioral test of the results and interpretations of Figures 3–6.

**FIGURE 7:**
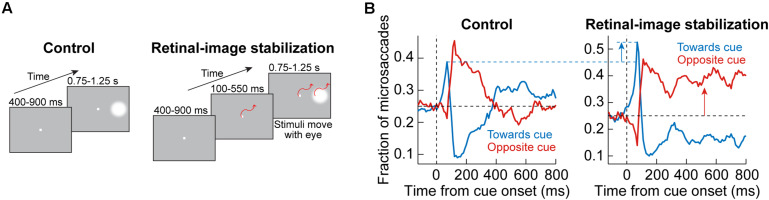
Behavioral causal manipulation to isolate the influences of SC and FEF activity on cue-induced microsaccades. **(A)** Behavioral task to experimentally control the spatial landscape of visual and oculomotor activity (Tian et al., 2016). In control (left), a peripheral cue appeared during fixation and was maintained throughout trial duration. With real-time retinal-image stabilization, the fixation spot and cue were experimentally pegged on the retina (that is, moved with the eye). Thus, a cue-induced early microsaccade did not generate a foveal position error at fixation that needed to be corrected; on the other hand, it could move the peripheral stimulus even farther out on the display (potentially rendering it invisible beyond the display edge if there was no top-down compensation that is implemented). **(B)** In control (left panel), a plot of microsaccade direction distributions reveals a similar oscillation to Figure 1: first, there was a bias of movements toward the cue (blue curve elevating above the horizontal black dashed line), and then there was a bias of opposite movements (red curve elevating above the horizontal black dashed line). With retinal-image stabilization (right panel), the early cue-induced effect was amplified. This was because the spatial layout of the peripheral stimulus onset was no longer competing with other factors like foveal position error at the fixation spot, which was uncontrolled in the control condition. Moreover, for subsequent microsaccades, there was a strong bias opposite the cue. Without such a bias, the peripheral stimulus could eventually have moved out of the display if all microsaccades continued to be toward the cue. Therefore, this causal manipulation further highlights the notion that post-inhibition microsaccades require top down strategic control, whereas early cue-induced microsaccades (during microsaccadic inhibition) are more reflexive (and likely subcortically mediated). Adapted with permission from Tian et al. (2016).

This approach was adopted by Tian et al. (2016, 2018). In their experiments, monkeys fixated, and a peripheral visual stimulus appeared and persisted on the display (Figure 7A). In control trials (Figure 7A, left), the normal visual-motor loop was active because the stimuli were stable on the display; therefore, whenever an eye movement happened, the visual error at the fixation spot was altered due to movement of the retinal image. On the other hand, in retinal-image stabilization experiments (Figure 7A, right), both the fixation spot and peripheral stimulus were presented in a gaze-contingent manner. Thus, the fixation spot and peripheral stimulus were rendered much more stable on the retina. Moreover, the fixation spot was stabilized as close as possible to the current gaze position, thus resulting in minimal visual error for most of the time (Tian et al., 2016).

In the control trials, oscillations in microsaccade directions after stimulus onset were evident (Figure 7B, left), as expected from Figure 1. However, with retinal-image stabilization, two key results emerged. First, the early cue-directed bias in microsaccade directions was still present, but it was amplified relative to control trials (blue upward arrow). This supports the mechanisms laid out in Figure 5. Specifically, without retinal image stabilization, the microsaccade goal (magenta location in Figure 5A) could either be congruent with the upcoming peripheral stimulus location (the situation depicted in Figure 5A) or it could be incongruent. This was simply uncontrollable because the peripheral stimulus always came asynchronously to ongoing state. Therefore, if it happened that the peripheral bursts occurred with a microsaccade goal being in the opposite direction, then there would have been a conflict between the need of the oculomotor system to correct the foveal error (by implementing the microsaccade burst) and the impact of peripheral visual bursts in the other direction. This is a condition that makes it harder for the peripheral cue to “attract” microsaccades (Tian et al., 2016, 2018). If the microsaccade goal happened to be congruent with the peripheral visual bursts, then cue-directed microsaccades were easier to trigger. As a result, on average, the effect was muted when compared to the retinal-image stabilization condition, in which the visual error at the fixation spot was experimentally minimized and controlled on every single trial (Figure 7B, blue upward arrow). Thus, not only does the SC contribute to modifying early microsaccade metrics in a seemingly reflexive manner (Figures 3–5), but this is functionally related to what the oculomotor system is anyway trying to achieve when gaze fixation is required: minimize visual errors at fixation (Tian et al., 2016).

The second effect that happened with retinal-image stabilization was the observation that subsequent microsaccades (after the initial cue-congruent movements) became much more strongly biased away from the persistent stimulus (Figure 7B, right; red upward arrow). Therefore, unlike the earlier cue-directed microsaccades, which were minimally affected, subsequent microsaccades became very different with a different kind of behavioral context (this time, the retinal-image stabilization context). Again, this supports the notion that post-inhibition microsaccades are a deliberate act relying on frontal cortical control (Figure 6), and therefore dependent on behavioral task. Indeed, if the eyes were to reflexively follow the cue continuously under retinal-image stabilization, then the peripheral target would end up moving beyond the extent of the visual display; success in the task required a purposeful strategy to bias microsaccades in the opposite direction to maintain view of the peripheral target on the display until trial end (Tian et al., 2016). If post-inhibition microsaccades were not under top-down control, then such contextual modification of the directional bias of these subsequent microsaccades would not be possible.

### Summary and Outlook

Microsaccadic modulations after peripheral cue onsets are stereotypical (Figure 1), but they have dissociable components in terms of their underlying mechanisms. Certain components of the modulations, such as microsaccadic inhibition, are unaccounted for by large perturbations of both the SC (Figure 2) and FEF (Figure 6). On the other hand, other components, such as directional biases, are separated based on when they happen: early biases are mediated by the SC and seem to be reflexive (Figures 3–5); later biases are mediated by the FEF (Figure 6) and seem to be deliberate. Functionally, all components of cue-induced microsaccadic modulations aim to optimize eye position at the fixation spot, despite momentary reflexive tendencies to be attracted by the suddenly appearing peripheral cues (Figure 7). This leaves a final unanswered question about the cue-induced microsaccadic modulations studied in the current article: why is microsaccadic inhibition so resilient to large inactivations of the SC and FEF, and what mediates it?

## The Primary Visual Cortex (V1)

Microsaccadic inhibition must have an inherently sensory component associated with it. First, it arrives at the time of cue-induced visual bursts (e.g., Figures 1, 5). Second, microsaccadic inhibition timing and strength depend on various stimulus properties, such as cue contrast, spatial frequency, and luminance contrast polarity (Rolfs et al., 2008; Bonneh et al., 2015; Scholes et al., 2015; Malevich et al., 2020). The inhibition is also correlated with subjective stimulus visibility (White and Rolfs, 2016). Therefore, even though the inhibition itself is a motor action, it must clearly be sensitive to visual sensory signals. This might suggest that an early sensory area, like V1, can contribute to microsaccadic inhibition, by virtue of its obvious sensory capabilities.

**FIGURE 8:**
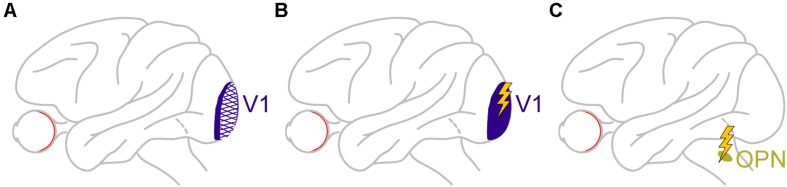
Causal manipulations in V1 and also downstream of the SC provide additional insights on the dissociable roles of cortical and subcortical pathways in mediating cue-induced influences on microsaccades. **(A)** Monkeys with large V1 lesions can perform cueing tasks (Yoshida et al., 2017), and how microsaccades in them are affected will provide a rich source of information on cortical routes for affecting cue-induced microsaccades. The available evidence so far suggests that, for at least some type of cues, V1 is not necessary for microsaccadic inhibition to occur (Yoshida and Hafed, 2017). **(B)** In monkeys with an intact V1, brief microstimulation pulses mimicking the brief visual bursts caused by cue onsets also provide hints on the role of V1 visual bursts in microsaccadic rate and direction modulations. In this case, V1 is sufficient for inhibition to occur (Buonocore et al., 2020a), likely through the generation of visual phosphenes that eventually propagate into the oculomotor system. **(C)** Finally, brief microstimulation pulses mimicking brief visual bursts (Buonocore et al., 2020a) in omnipause neurons (OPN’s), downstream of the SC, are isolating a role for these neurons, which constitute the final gating point for saccade generation, in implementing microsaccadic inhibition. The lack of influence of SC and FEF inactivation on microsaccadic inhibition (Figures 2, 6), as well as the persistence of inhibition even with V1 lesions (for at least the types of cues tested so far), might mean that it is indeed OPN’s that are the most critical structure for implementing microsaccadic inhibition.

In another large perturbation experiment, large portions of unilateral V1 were lesioned in monkeys (Figure 8A), in order to generate an animal model of blindsight (Yoshida et al., 2008, 2012; Isa and Yoshida, 2009; Ikeda et al., 2011; Kato et al., 2011; Takaura et al., 2011). In addition to all of the characterizations of visual and oculomotor capabilities of these animals in the above studies, it was recently found that these “blindsight monkeys” could also still perform covert cueing tasks, albeit with altered performance (Yoshida et al., 2017). This finding was important because it represented an excellent opportunity to test for a causal role of V1 in microsaccadic inhibition (and other modulations). Therefore, the authors analyzed microsaccades in these animals during cueing tasks. Preliminary results so far (Yoshida and Hafed, 2017) reveal that microsaccadic inhibition still took place, despite the large V1 lesions, although the analyses were made with foveal cues (foveal V1 was largely spared by the lesions). It therefore remains to be seen how microsaccadic inhibition in these animals behaved when peripheral cues, like in the tasks of Peel et al. (2016), were used. It is highly likely, in our opinion, that microsaccadic inhibition will still be seen, suggesting that an intact V1 is not necessary for microsaccadic inhibition to occur. Interestingly, there were other asymmetries in microsaccade generation that resulted from V1 lesions, but these are beyond the scope of this exposition.

The fact that V1 might not be necessary for microsaccadic inhibition does not mean that V1 cannot still contribute, at least indirectly. After all, cue onsets are expected to cause short-lived visual bursts in V1, and at roughly similar times to the visual bursts in the SC (e.g., Figure 5). Moreover, signals from V1 bursts can then propagate, with short latencies, to areas that can ultimately influence the oculomotor system. To test for this idea, in yet additional preliminary perturbation results (Buonocore et al., 2020a), Buonocore et al. recently electrically microstimulated V1 neurons with very brief bursts of pulses (Figure 8B). These brief pulse trains were intended to simulate the occurrence of visually-driven neural bursts after visual stimulus onsets. The monkeys simply fixated a spot, and brief bursts of microstimulation pulses were injected into V1. Shortly after microstimulation onset, microsaccade rate was indeed modulated in a manner very similar to that in Figure 1A. The primary difference was that the inhibition started even earlier than in Figure 1A, and the rebound also came earlier (Buonocore et al., 2020a). This is consistent with V1 microstimulation inducing a visual phosphene (Tehovnik et al., 2005; Schiller et al., 2011; Chen et al., 2020) that essentially bypassed the delay of the retino-cortical pathway associated with a normal visual stimulus impinging on the retina. Therefore, the whole curve of Figure 1A was just shifted backwards in time. This means that even though V1 is not necessary for microsaccadic inhibition to occur, based on the preliminary lesion data of Yoshida and Hafed (2017), it is sufficient for the inhibition to be seen, as evident from the preliminary microstimulation data (Buonocore et al., 2020a). Such sufficiency is probably mediated by signal propagation of V1-induced phosphenes to normal pathways eventually affecting the oculomotor system.

We are thus back to square one with respect to discovering the primary source for microsaccadic inhibition in Figure 1A: even with large V1 perturbation through lesioning, microsaccadic inhibition seems to be still intact.

## The Need for Additional Subcortical Mechanisms in Mediating Cue-Induced Modulations of Microsaccades

To complete the search for a potential brain area that is both necessary and sufficient for microsaccadic inhibition, it was necessary to start explicitly testing an earlier hypothesis (Hafed and Ignashchenkova, 2013; Buonocore et al., 2017) that microsaccadic inhibition needs to be mediated by a brain region that is both sensitive to visual inputs and also capable of rapidly changing the likelihood to generate a saccade. This hypothesis has directly motivated studying a class of neurons in the nucleus raphe interpositus (rip) in the lower brainstem, and downstream of the SC. These neurons are called omnipause neurons (OPN’s), and they derive their name from a very distinctive property: the neurons are tonically active, and they only completely pause their activity during any saccade of any size and any direction (Cohen and Henn, 1972; Luschei and Fuchs, 1972; Keller, 1974; Missal and Keller, 2002). OPN’s are thus thought to act as the final gating point to allow saccades to happen (Cohen and Henn, 1972; Luschei and Fuchs, 1972; Keller, 1974; Gandhi and Keller, 1999; Missal and Keller, 2002), and electrically microstimulating OPN’s in the middle of saccades is sufficient to interrupt the movements mid-flight (Keller et al., 1996; Gandhi and Keller, 1999). These neurons thus satisfy one of the two criteria for successfully implementing microsaccadic inhibition: the neurons should be capable of changing the likelihood of microsaccades by changing whether they remain tonically active or pause.

However, as stated above, microsaccadic inhibition must also be sensitive to sensory stimuli, and it was not known, so far, whether OPN’s can exhibit sophisticated sensory tuning properties to image characteristics like contrast, spatial frequency, and orientation, which are all characteristics that can influence microsaccadic inhibition. It, therefore, became necessary to investigate whether OPN’s exhibit sophisticated visual pattern analysis capabilities, despite being the final motor gate for triggering saccades. Surprisingly, preliminary data revealed exactly such visual pattern analysis capabilities (Buonocore et al., 2020a). Thus, OPN’s satisfy the two criteria for mediating rapid microsaccadic inhibition: sensitivity to visual stimuli of different patterns from the outside world, and an ability to gate saccades (and microsaccades) with very short latencies.

To further test this hypothesis, Buonocore et al. then started electrically microstimulating OPN’s during steady-state fixation (Figure 8C), much like they did in V1. Brief pulse trains were introduced to mimic short-lived visual bursts by these neurons. Microsaccade rate was reduced with even shorter latencies than with V1 microstimulation, and there was no appreciable microsaccadic rebound afterward (Buonocore et al., 2020a). Interestingly, brief pulse trains in the SC, to mimic SC visual bursts, instead increased microsaccade likelihood rather than decreased it, and there was a strong directional and amplitude component as well (directly consistent with the results of Figure 5).

Therefore, in all likelihood, microsaccadic inhibition was so resilient to inactivation of the SC and FEF (and lesioning of V1) simply because it is a phenomenon that is critically dependent on yet another brain area, even more downstream of the SC. In our opinion, this area is the rip, containing OPN’s.

## An Integrative View of Currently Known Cortical and Subcortical Circuits Mediating the Influences of Visual Cues on Microsaccades

**FIGURE 9:**
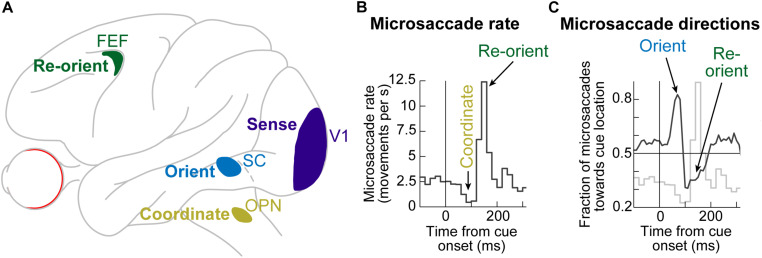
An integrative view of the cortical and subcortical contributions to modulations of microsaccades after visual cues. **(A)** Cue onsets are essentially “sensed” by all shown areas. However, the visual bursts occurring in the different areas contribute differential roles. Visual bursts in V1 likely serve visual detection in general. However, those in the SC at very similar times influence microsaccade directions, and those in OPN’s (again at similar times) influence coordination of microsaccade timing to result in microsaccadic inhibition. Such early cue-induced visual bursts in all of these areas likely trump the influences of early visual bursts in FEF, because the FEF seems to be least critical for early cue-induced microsaccades (Figure 6). Rather, FEF activity matters much more after the initial reflexive influences mediated subcortically. Thus, FEF activity serves to re-orient microsaccades after the initial cue-induced reflexes. **(B)** The integrative view in **(A)** allows explicitly interpreting the individual components of known modulations in microsaccades after cue onset (e.g., Figure 1). In terms of microsaccade rate, visual bursts in OPN’s allow coordination of microsaccade timing, resulting in microsaccadic inhibition. Subsequent microsaccades (during the post-inhibition rebound phase) are mediated by FEF re-orienting. **(C)** In terms of microsaccade direction oscillations, SC visual bursts after cue onset are read out in a way to influence microsaccade directions toward the appearing cues. Subsequent microsaccades are deliberate efforts to maintain the eye at the fixated target despite the peripherally appearing cue. Therefore, microsaccade direction oscillations reflect an initial reflexive orientation mediated by the SC followed by a deliberate re-orientation mediated by the FEF.

Taking all of the above evidence together, one can now develop an integrative view of the currently known cortical and subcortical circuits responsible for the stereotypical cue-induced microsaccadic modulations of Figure 1. The SC may be viewed as critical for reflexive orienting responses by early cue-induced microsaccades, whereas the FEF serves a re-orienting purpose to influence subsequent deliberate movements (Figure 9A). In terms of microsaccade timing, V1 senses peripheral stimuli, but it only influences microsaccadic inhibition indirectly, or at least less directly than OPN’s, which can help to coordinate microsaccade timing much more precisely (by rapidly inhibiting movements after cue onsets). Finally, in terms of the actual modulations of microsaccade rates and directions, Figures 9B,C now adds labels of the mechanisms associated with the brain areas in Figure 9A for the specific components of the so-called microsaccadic rate signature after cue onsets (Figure 9B) and the related time course of microsaccade direction oscillations (Figure 9C). While further investigations of V1 and OPN’s are needed in order to solidify the emerging picture, the scheme laid out in Figure 9 provides an important foundation for understanding the functional implications of microsaccades in covert visual selection tasks. Additional investigations of other visual patterns for the cues, as well as additional visual, cognitive, and motor pathways, will become necessary to develop an even more complete picture, for example, with respect to other cognitive factors that can influence microsaccades (such as memory, reward, motivation, and fatigue).

## Conclusion

In this work, we reviewed the causal perturbation evidence for explaining highly robust modulations of microsaccadic eye movements after peripheral cueing. We particularly described dissociable contributions to both microsaccade likelihood and microsaccade direction from different cortical and subcortical regions, like the SC, FEF, and V1. In the future, additional insights can be gleaned when combining behavioral perturbation manipulations, such as in Figure 7, with either neurophysiological recordings or neurophysiological perturbation manipulations. In all, we believe that studying the neural mechanisms for the influences of cues on microsaccades can illuminate broader questions on the links between perception, cognition, and action, and in multiple species as well. The links between microsaccades and covert visual selective mechanisms remain to be a highly interesting topic of investigation.

## Author Contributions

All authors listed have made a substantial, direct and intellectual contribution to the work, and approved it for publication.

## Conflict of Interest

The authors declare that the research was conducted in the absence of any commercial or financial relationships that could be construed as a potential conflict of interest.
